# Selective reconstitution of IFN‑γ gene function in Ncr1^+^ NK cells is sufficient to control systemic vaccinia virus infection

**DOI:** 10.1371/journal.ppat.1008279

**Published:** 2020-02-05

**Authors:** Katharina Borst, Sven Flindt, Patrick Blank, Pia-Katharina Larsen, Chintan Chhatbar, Jennifer Skerra, Julia Spanier, Christoph Hirche, Martin König, Tomas Alanentalo, Martin Hafner, Zoe Waibler, Klaus Pfeffer, Veronika Sexl, Gerd Sutter, Werner Müller, Theresa Graalmann, Ulrich Kalinke

**Affiliations:** 1 TWINCORE–Centre for Experimental and Clinical Infection Research, Institute for Experimental Infection Research, Hanover, Germany; 2 Paul-Ehrlich-Institut, Division of Immunology, Langen, Germany; 3 European Molecular Biology Laboratory (EMBL), Mouse Biology Programme, Monterodonto, Italy; 4 Helmholtz Centre for Infection Research, Brunswick, Germany; 5 University of Düsseldorf, Institute of Medical Microbiology and Hospital Hygiene, Düsseldorf, Germany; 6 University of Veterinary Medicine (VetmedUni), Institute for Pharmacology and Toxicology, Vienna, Austria; 7 LMU University of Munich, Institute for Infectious Diseases and Zoonoses, Munich, Germany; 8 Clinic for Immunology and Rheumathology, Hannover Medical School, Carl-Neuberg-Straße 1, 30625 Hannover, Germany; 9 Cluster of Excellence—Resolving Infection Susceptibility (RESIST) (EXC 2155), Hannover Medical School, Carl-Neuberg-Straße 1, 30625 Hannover, Germany; Harvard Medical School, UNITED STATES

## Abstract

IFN-γ is an enigmatic cytokine that shows direct anti-viral effects, confers upregulation of MHC-II and other components relevant for antigen presentation, and that adjusts the composition and balance of complex cytokine responses. It is produced during immune responses by innate as well as adaptive immune cells and can critically affect the course and outcome of infectious diseases, autoimmunity, and cancer. To selectively analyze the function of innate immune cell-derived IFN-γ, we generated conditional IFN-γ^OFF^ mice, in which endogenous IFN-γ expression is disrupted by a loxP flanked gene trap cassette inserted into the first intron of the IFN-γ gene. IFN-γ^OFF^ mice were intercrossed with Ncr1-Cre or CD4-Cre mice that express Cre mainly in NK cells (IFN-γ^Ncr1-ON^ mice) or T cells (IFN-γ^CD4-ON^ mice), respectively. Rosa26RFP reporter mice intercrossed with Ncr1-Cre mice showed selective RFP expression in more than 80% of the NK cells, while upon intercrossing with CD4-Cre mice abundant RFP expression was detected in T cells, but also to a minor extent in other immune cell subsets. Previous studies showed that IFN-γ expression is needed to promote survival of vaccinia virus (VACV) infection. Interestingly, during VACV infection of wild type and IFN-γ^CD4-ON^ mice two waves of serum IFN-γ were induced that peaked on day 1 and day 3/4 after infection. Similarly, VACV infected IFN-γ^Ncr1-ON^ mice mounted two waves of IFN-γ responses, of which the first one was moderately and the second one profoundly reduced when compared with WT mice. Furthermore, IFN-γ^Ncr1-ON^ as well as IFN-γ^CD4-ON^ mice survived VACV infection, whereas IFN-γ^OFF^ mice did not. As expected, *ex vivo* analysis of splenocytes derived from VACV infected IFN-γ^Ncr1-ON^ mice showed IFN-γ expression in NK cells, but not T cells, whereas IFN-γ^OFF^ mice showed IFN-γ expression neither in NK cells nor T cells. VACV infected IFN-γ^Ncr1-ON^ mice mounted normal cytokine responses, restored neutrophil accumulation, and showed normal myeloid cell distribution in blood and spleen. Additionally, in these mice normal MHC-II expression was detected on peripheral macrophages, whereas IFN-γ^OFF^ mice did not show MHC-II expression on such cells. In conclusion, upon VACV infection Ncr1 positive cells including NK cells mount two waves of early IFN-γ responses that are sufficient to promote the induction of protective anti-viral immunity.

## Author summary

Viral infections induce interferon (IFN) responses that constitute a first line of defense. Type II IFN (IFN-γ) is required for protection against lethal vaccinia virus (VACV) infection. To address the cellular origin of protective IFN-γ responses during VACV infection, we generated IFN-γ^OFF^ mice, in which the endogenous IFN-γ gene function can be reconstituted in a Cre-dependent manner. IFN-γ^OFF^ mice were intercrossed with Ncr1-Cre mice that express Cre selectively in Ncr1^+^ innate cell subsests such as NK cells. Surprisingly, VACV infected IFN-γ^Ncr1-ON^ mice mounted two waves of IFN-γ responses. Reconstitution of innate IFN-γ was sufficient to restore cytokine responses that supported normal myeloid cell distribution and survival upon VACV infection. In conclusion, IFN-γ derived from Ncr1^+^ innate immune cells is sufficient to elicit fully effective immune responses upon VACV infection. Our new mouse model is suitable to further address the role of Ncr1^+^ cell-derived IFN-γ also in other models of infection, as well as of autoimmunity and cancer.

## Introduction

Upon viral infection, interferons play a crucial role in host protection. While type I interferons (IFN-I) primarily confer early anti-viral effects, type II interferon (IFN-γ) additionally activates myeloid cells, and induces Th1 driven adaptive immunity [1, 2]. IFN-γ is expressed by innate immune cells such as NK cells as well as by adaptive immune cells such as T cells. NK cells rapidly react to viral infections by lysing infected cells directly in an antigen-independent manner and by producing cytokines such as IFN-γ until the adaptive immune system is sufficiently activated to control the infection [3–9]. Also stimulated CD4^+^ and CD8^+^ T cells produce IFN-γ and they are of key relevance for the induction of long-term memory responses. So far, the role of innate and adaptive immune cell-derived IFN-γ expression was addressed by selective depletion of specific cell subsets or by adoptive transfer experiments [10–15]. However, the particular role of innate immune cell-derived IFN-γ expression has not yet been addressed specifically.

Live vaccinia virus (VACV) vaccination was successfully used to eradicate the infectious agent of the human smallpox disease, variola virus [16]. VACV has been extensively used as a model to study the induction and effector mechanisms of early innate as well as adaptive immunity. Upon VACV infection, IFN-γ deficient (IFN-γ^-/-^) and IFN-γ receptor deficient (IFN-γR^-/-^) mice show enhanced susceptibility to lethal disease [17, 18]. VACV infection induces early IFN**-**I responses [19], which activate myeloid cells to produce cytokines such as IL-15 and IL-12/IL-18 that subsequently activate NK cells [10, 20–24]. Activated NK cells are essential to control VACV replication and are capable to produce IFN-γ [21, 23, 25, 26]. Early IFN-γ production may then induce MHC-II expression on antigen presenting cells (APC), which is crucial for CD4^+^ T cell activation [27–29]. So far, there have been inconsistent data on the importance of CD4^+^ T-cell help during VACV infection [11–13]. Nevertheless, adaptive immune responses are needed to control VACV infection since VACV infected RAG^-/-^ mice are rescued by adoptive transfer of IFN-γ producing CD8^+^ T cells [14, 15, 18, 25, 30]. Further dissection of the role of innate immune cell-derived IFN-γ expression during the initial phase of viral infections is needed for the development of new vaccination strategies that induce protective immunity.

To specifically analyze the role of early innate immune cell-derived IFN-γ expression during viral infection, we generated conditional IFN-γ^OFF^ mice, in which the IFN-γ gene function is disrupted and can be reconstituted in a Cre-dependent manner. We intercrossed IFN-γ^OFF^ mice with Nrc1-Cre^+/-^ mice, expressing Cre mainly in NK cells, and CD4-Cre^+/-^ mice, expressing Cre mainly in T cells. Our results verified earlier observations that Nrc1-Cre showed highly cell type selective Cre expression, whereas CD4-Cre mice showed Cre expression also in other cell subsets than T cells and therefore were not optimally suited for Cre-dependent gene reconstitution approaches. Interestingly, in IFN-γ^Ncr1-ON^ mice the cell sective IFN-γ gene reconstitution was sufficient to balance cytokine responses, induce myeloid cell accumulation, control viral replication, and to clear the VACV infection.

## Results

### Mice with a Ncr1-specific reconstitution of the IFN-γ gene function survive VACV infection

**Fig 1:**
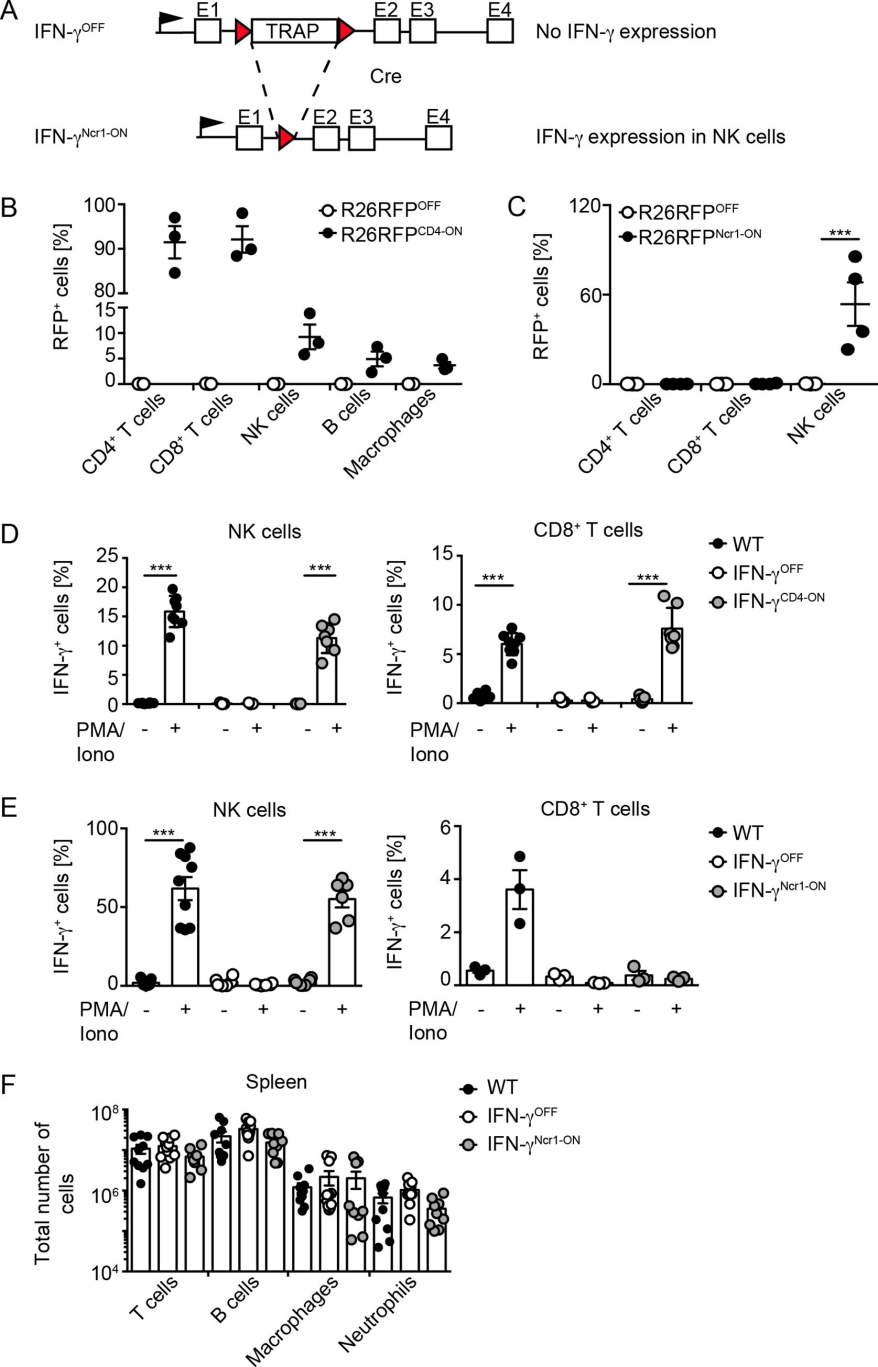
Cell selectivity of Cre-mediated recombination using CD4-Cre and Ncr1-Cre mice. (A) Schematic depiction of TRAP-mediated inactivation of IFN-γ and of Cre-mediated reconstitution of the *Ifng* gene function. (B) Splenocytes of R26RFP^OFF^ (white) and R26RFP^CD4-ON^ mice (black) were isolated and immune cell subsets were analyzed by flow cytometry for RFP expression (n = 3, N = 1), one-tailed Mann-Whitney U test. (C) Splenocytes of R26RFP^OFF^ (white) and R26RFP^Ncr1-ON^ mice (black) were isolated and T and NK cells were analyzed by flow cytometry for RFP expression (n ≥ 3, N = 2), one-tailed Mann-Whitney U test. (D) Splenocytes were isolated from WT, IFN-γ^OFF^ and IFN-γ^CD4-ON^ mice, stimulated with PMA/ionomycin for 4 h, and then analyzed by flow cytometry (n ≥ 3, N = 2), paired T-test. Percentage of IFN-γ expressing T or NK cells is shown. Error bars indicate mean ± SEM; ***p ≤ 0.001. (E) Splenocytes were isolated from WT, IFN-γ^OFF^ and IFN-γ^Ncr1-ON^ mice, stimulated with PMA/ionomycin for 4 h, and then analyzed by flow cytometry (n ≥ 3, N = 1–3), paired T-test. Percentage of IFN-γ expressing T or NK cells is shown. Error bars indicate mean ± SEM; ***p ≤ 0.001. (F) Immune cells were isolated from naïve WT, IFN-γ^OFF^ and IFN-γ^Ncr1-ON^ mice and total numbers of T cells, B cells, macrophages, and polymorphonuclear neutrophils (PMN) in spleen was analyzed by FACS (n ≥ 9, N = 3), one-tailed Mann-Whitney U test.

To address the function of innate immune cell-derived IFN-γ, we generated IFN-γ^OFF^ mice that carry a floxed gene trapping cassette (TRAP) in the first intron of the IFN-γ gene and thus lack IFN-γ expression (for schematic depiction see Fig 1A). To selectively reconstitute the IFN-γ gene function in T cells or NK cells, we planned to intercross IFN-γ^OFF^ mice with CD4-Cre mice that show Cre expression primarily in T cells ([31], IFN-γ^CD4-ON^ mice) or Ncr1-Cre mice that show NK cell-specific Cre expression ([32], IFN-γ^Ncr1-ON^ mice), respectively. To test the cell subset-specificity of the Cre expression first, we intercrossed CD4-Cre and Ncr1-Cre mice with Rosa26RFP reporter mice and determined RFP expression in various cell subsets as a measure of Cre-mediated recombination by flow cytometry. Of note, in CD4-Cre^+^Rosa26RFP mice RFP expression was detected primarily in T cells, but to a minor extent also in certain innate immune cell subsets such as NK cells, some of which may express CD4 (Fig 1B). In contrast, in Ncr1-Cre^+^Rosa26RFP mice more than 50% of conventional Nk1.1^+^Ncr1^+^ NK cells, which are also classified as Ncr1^+^ ILC1 [33, 34], showed RFP expression (Fig 1C). Of note, amongst non-NK ILC1, which represent a very rare cell subset within the liver, only 1-2% of the cells showed marginal RFP expression (S1 Fig).

To next study the cell-selective reconstitution of the IFN-γ gene function, spleen cells were *in vitro* treated with PMA/ionomycin and intracellular IFN-γ expression was determined by flow cytometry analysis. Indeed, under such conditions splenocytes from WT mice showed IFN-γ expression in T cells as well as in NK cells. Similarly, splenocytes from IFN-γ^CD4-ON^ mice showed IFN-γ expression in T cells and NK cells at a similar level as detected in splenocytes from WT animals (Fig 1D). In contrast, splenocytes from IFN-γ^OFF^ mice lacked intracellular IFN-γ expression, whereas splenocytes from IFN-γ^Ncr1-ON^ mice showed IFN-γ expression only in NK cells, but not in T cells (Fig 1E). Thus, IFN-γ^Ncr1-ON^ mice are suitable to study the *in vivo* function of innate cell-derived IFN-γ responses. Since the group of NK cells comprises various cell subsets, we further analyzed NK cell-specific IFN-γ gene reconstitution in tissue-resident NK cells and conventional NK cells. To this end, lymphocytes were isolated from liver of WT, IFN-γ^OFF^, and IFN-γ^Ncr1-ON^ mice and *in vitro* treated with PMA/ionomycin. Interestingly, only conventional NK cells mounted IFN-γ responses in WT and IFN-γ^Ncr1-ON^ mice, while liver-resident NK cells barely expressed IFN-γ (S1 Fig).

**Fig 2:**
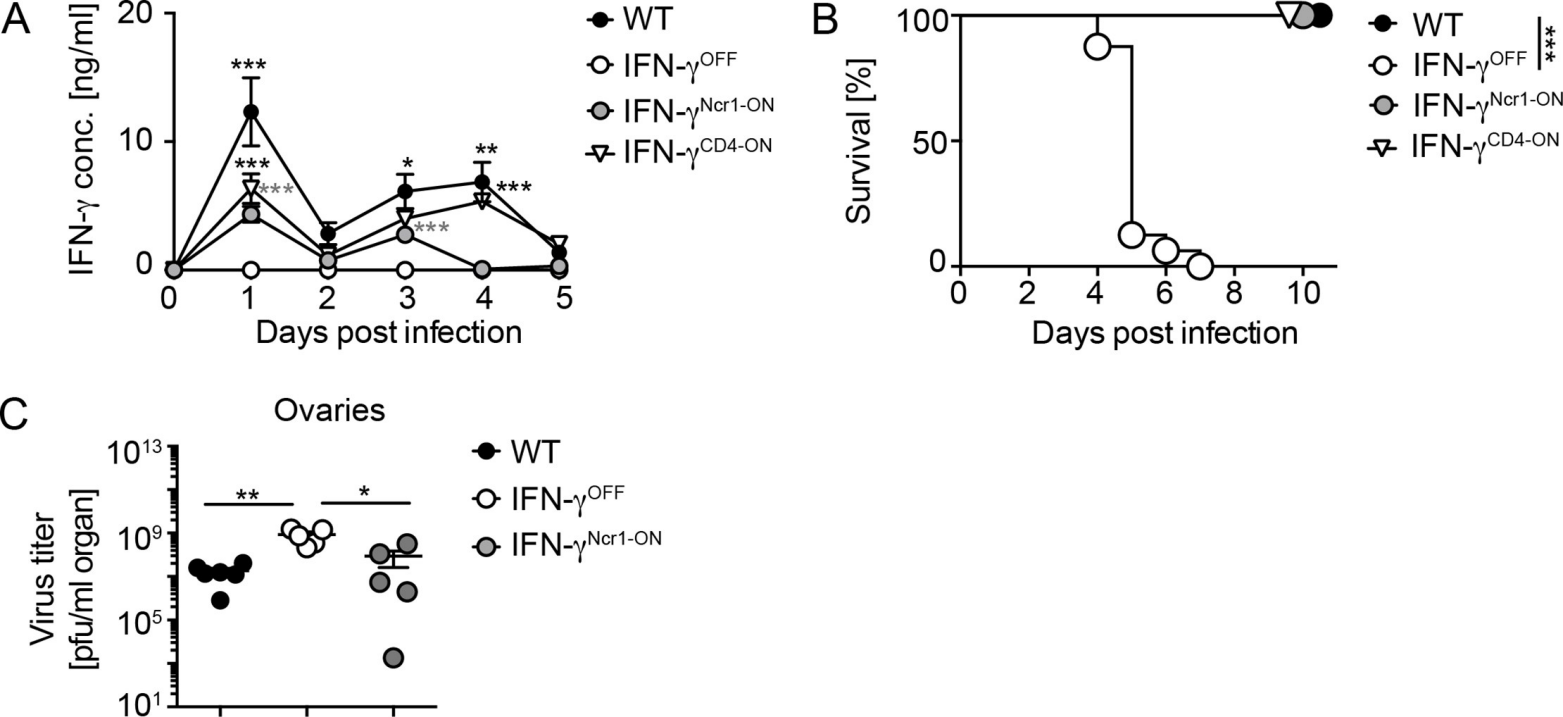
IFN-γ^Ncr1-ON^ mice are protected against lethal VACV infection. WT, IFN-γ^OFF^, IFN-γ^CD4-ON^ and IFN-γ^Ncr1-ON^ mice were i.v. infected with 2 x 10^6^ pfu VACV. (A) Serum samples were drawn at the indicated time points and analyzed for the IFN-γ content by an ELISA method (n = 6, N = 2); one-way Anova. (B) Survival was monitored and in case body weight decrease by more than 20% of the initial bodyweight, or when the overall health status was dramatically reduced, mice were sacrificed (n ≥ 10, N = 3); Mantel Cox test. (C) Virus loads in ovaries was determined 5 days post infection (dpi) by plaque assay (n ≥ 5, N = 2). Error bars indicate mean ± SEM; ***p ≤ 0.001, **p ≤ 0.01, *p ≤ 0.05; one-tailed Mann-Whitney U test.

As expected from previous studies with conventional IFN-γ^-/-^ and IFN-γR^-/-^ mice [17, 35], under homeostatic conditions IFN-γ^OFF^ as well as IFN-γ^Ncr1-ON^ mice showed an overall normal immune cell distribution in the spleen (Fig 1F). To study whether upon viral infection CD4- or Ncr1-specific IFN-γ gene reconstitution affects systemic IFN-γ levels, WT, IFN-γ^OFF^, IFN-γ^CD4-ON^ and IFN-γ^Ncr1-ON^ mice were intravenously (i.v.) infected with 2 x 10^6^ pfu vaccinia virus (VACV), serum samples were drawn at the indicated time points, and IFN-γ protein levels were determined by an ELISA method. In WT and IFN-γ^CD4-ON^ mice two waves of IFN-γ responses were detected, peaking on day 1 and 4 post infection (dpi) (Fig 2A). In contrast, IFN-γ^OFF^ mice lacked IFN-γ responses entirely (Fig 2A). Infected IFN-γ^Ncr1-ON^ mice also mounted two waves of IFN-γ, of which the first and the second one peaked on 1 and 3 dpi, respectively (Fig 2A). The overall magnitude of these Ncr1^+^ cell-derived IFN-γ responses was reduced when compared with WT mice on both days. This indicates that early after VACV infection (day 1 to 3) IFN-γ responses are contributed to a large extent by Ncr1^+^ innate immune cells, whereas at later time points IFN-γ is produced primarily by other cells such as T cells. Next, we analyzed whether the IFN-γ gene reconstitution in Ncr1^+^ cells suffices to promote survival upon VACV infection. Indeed, IFN-γ^Ncr1-ON^ mice survived the infection as well as WT and IFN-γ^CD4-ON^ mice without signs of severe disease, while IFN-γ^OFF^ mice succumbed to the infection within 5 days (Fig 2B). Furthermore, IFN-γ^Ncr1-ON^ mice were able to control viral replication, while IFN-γ^OFF^ mice showed highly elevated virus titers and succumbed to the infection (Fig 2C).

### Selective IFN-γ reconstitution only in Ncr1^+^ cells is sufficient to restore VACV-induced cytokine responses

**Fig 3:**
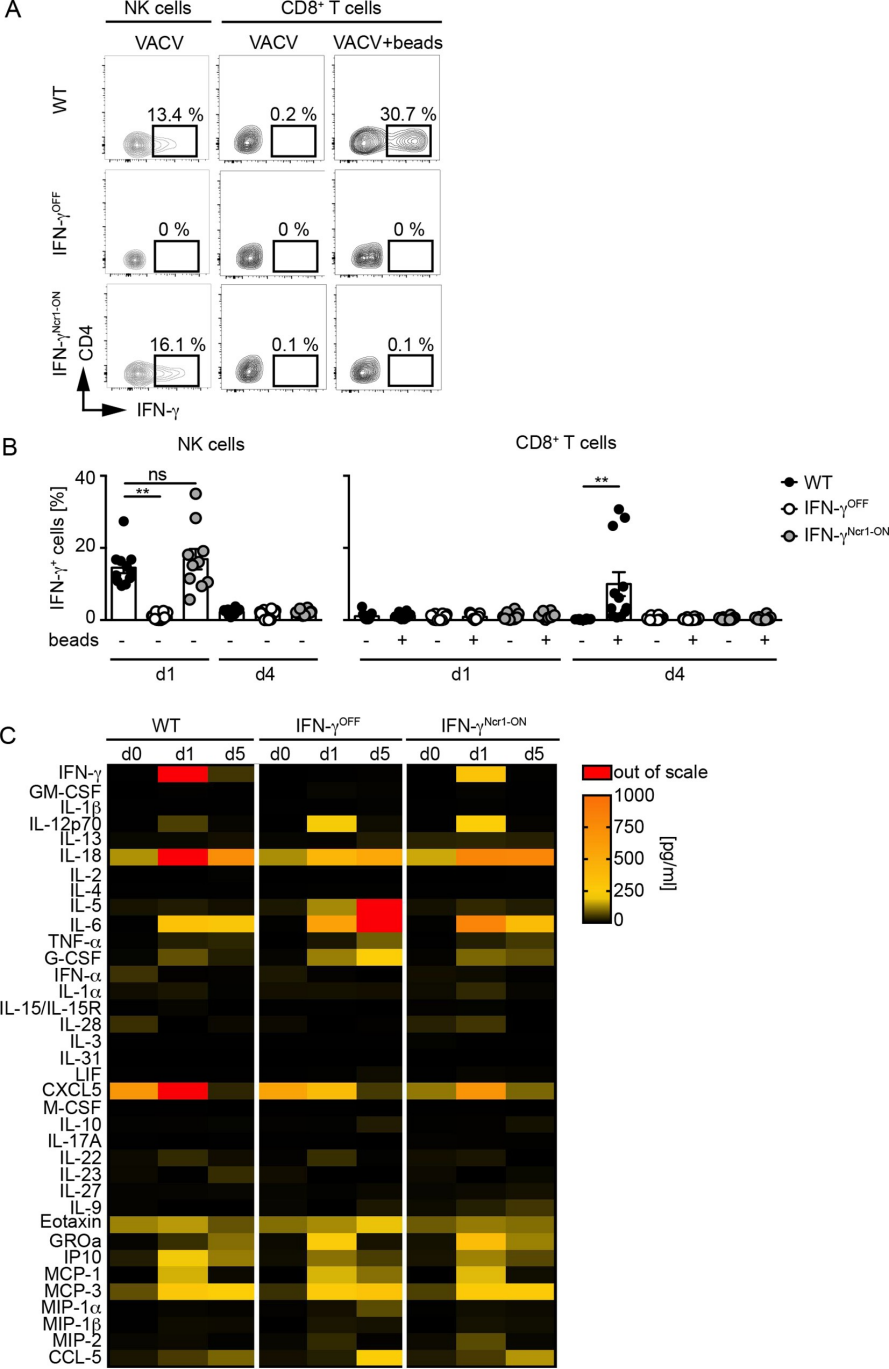
Balanced cytokine responses in VACV infected IFN-γ^Ncr1-ON^ mice. WT, IFN-γ^OFF^ and IFN-γ^Ncr1-ON^ mice were i.v. infected with 2 x 10^6^ pfu VACV. (A/B) Splenocytes of infected mice were isolated 1 and 4 dpi, *in vitro* stimulated with CD3/CD28 beads for 4 h and then analyzed by flow cytometry for IFN-γ expressing NK or T cells (n ≥ 10, N = 3); one-tailed Mann-Whitney U test. Error bars indicate mean ± SEM; ***p ≤ 0.001, **p ≤ 0.01, *p ≤ 0.05. (C) Serum samples were drawn at the indicated time points and analyzed with a multiplex cytokine and chemokine array (n = 3, N = 1).

To study the cellular source of innate IFN-γ in greater detail, WT, IFN-γ^OFF^, and IFN-γ^Ncr1-ON^ mice were VACV infected, 1 and 4 dpi splenocytes were isolated, and intracellular IFN-γ expression was analyzed by flow-cytometry in NK cells and T cells. *Ex viv*o isolated splenic NK cells of infected WT and IFN-γ^Ncr1-ON^ mice showed spontaneous intracellular IFN**-**γ expression, while in NK cells of IFN-γ^OFF^ mice no IFN-γ expression was detected (Fig 3A and 3B). Of note, 4 dpi IFN-γ expression was not detected in NK cells, irrespective of whether splenocytes from WT, IFN-γ^OFF^, or IFN-γ^Ncr1-ON^ mice were analyzed (Fig 3A and 3B). One dpi re-stimulated T cells lacked IFN-γ expression, irrespective of the genotype of the mice analyzed (Fig 3A and 3B). In contrast, 4 dpi only re-stimulated T cells from WT mice showed IFN-γ expression, while T cells from IFN-γ^OFF^ and IFN-γ^Ncr1-ON^ mice did not (Fig 3A and 3B). These results support the hypothesis that upon VACV infection early IFN-γ responses are conferred by Ncr1^+^ innate immune cells, while 4 dpi other cells, such as T cells, contribute to IFN-γ production.

To study effects of innate immune/NK cell-derived IFN-γ responses on the overall chemokine and cytokine milieu, serum of VACV infected WT, IFN-γ^OFF^, and IFN-γ^Ncr1-ON^ mice was analyzed 1 dpi, which is the peak of NK cell-derived IFN-γ responses, and at day 5, which is the terminal time point of IFN-γ^OFF^ mice, by a multiplex cytokine array. Already 1 dpi, enhanced cytokine levels were detected in the serum of infected WT mice, including IL-12p70, IL-18, and IL-6, which are known to be produced mainly by myeloid cells. Furthermore, chemokines, which regulate myeloid cells, such as MCP-1, MCP-3 and CXCL5 were induced (Fig 3C). By 5 dpi, the inflammatory cytokine response was already reduced in WT mice. Interestingly, in infected IFN-γ^OFF^ mice the overall cytokine milieu differed extensively from that of infected WT mice. Specifically, IL-18 and CXCL5 levels were decreased, while IL-12p70 was increased 1 dpi when compared with WT mice. On day 5 post infection the T_h_2 cytokine IL-5 and the pro-inflammatory cytokine IL**-**6 were massively upregulated in IFN-γ^OFF^ mice as well as G-CSF and CCL-5 when compared with infected WT controls. In IFN-γ^Ncr1-ON^ mice the overall cytokine and chemokine profile was reminiscent of that of WT mice, while the overall concentration of serum IFN-γ was reduced. Thus, IFN-γ expression by Ncr1^+^ cells is sufficient to balance VACV-induced cytokine and chemokine responses.

### IFN-γ expression of Ncr1^+^ cells regulates the distribution of peripheral myeloid cell subsets

**Fig 4:**
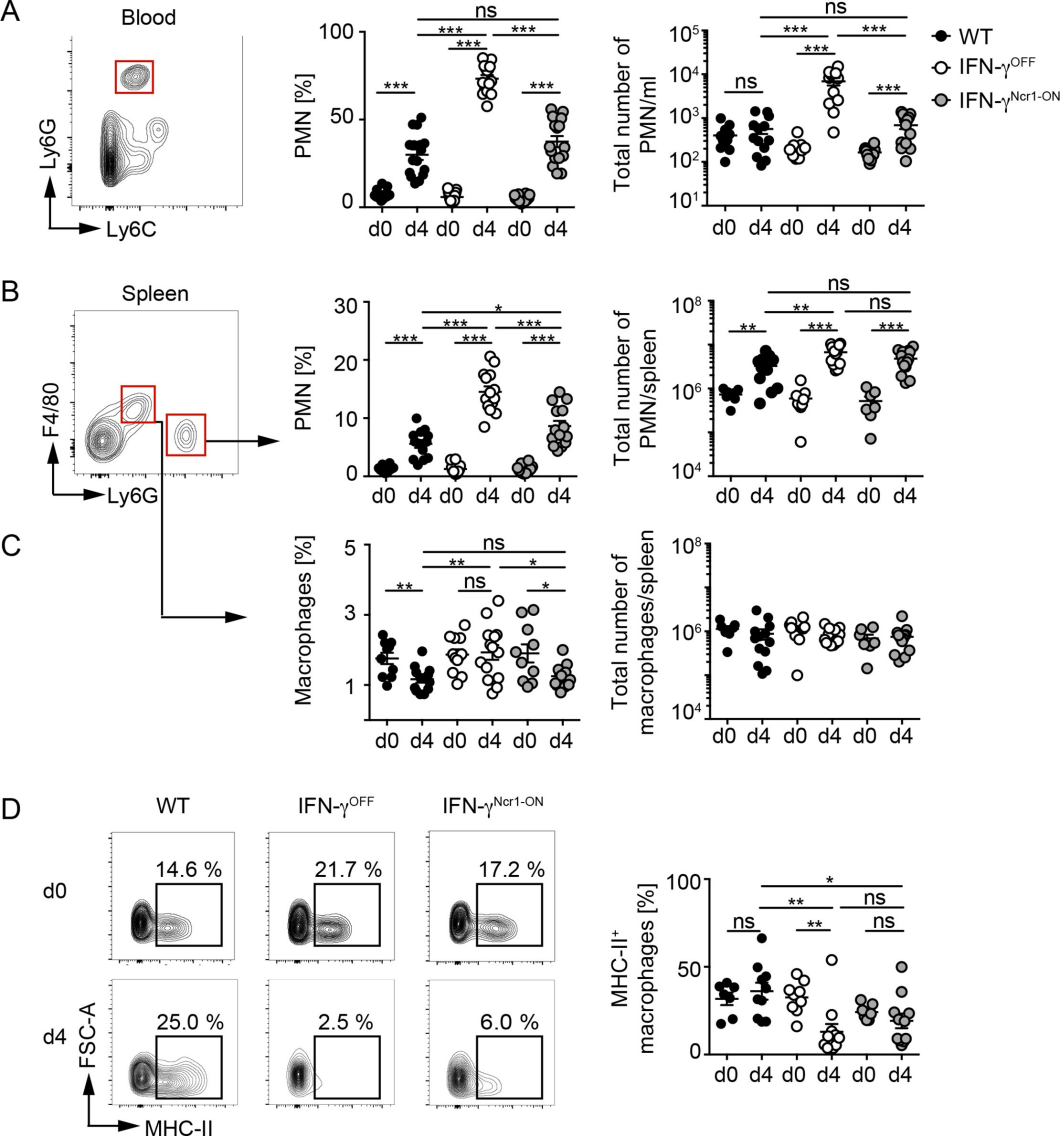
Normalized distribution of peripheral myeloid cell subsets in VACV infected IFN-γ^Ncr1-ON^ mice. WT, IFN-γ^OFF^ and IFN-γ^Ncr1-ON^ mice were i.v. infected with 2 x 10^6^ pfu VACV. At the indicated time points blood samples were drawn, spleen was prepared and myeloid cells were analyzed by flow cytometry (n ≥ 7, N = 3). (A) Percentages and total cell numbers of polymorphonuclear neutrophils (PMN) in the blood. Percentages and total cell numbers of (B) PMN or (C) macrophages in spleen (pregated on CD3^-^, CD19^-^ cells). (D) Percentages of MHC-II expressing macrophages in the spleen.

We next analyzed whether the early innate immune cell-derived IFN-γ affected the presence and activation of myeloid cells on 4 dpi. VACV-infected WT mice showed significantly increased percentages of polymorphonuclear neutrophils (PMN) in the blood 4 dpi when compared with uninfected controls (Fig 4A). These effects were even more pronounced in infected IFN-γ^OFF^ mice that had a significant increase of total numbers of PMN within the blood, while in infected IFN-γ^Ncr1-ON^ mice the percentages of PMN were comparable with those in WT mice (Fig 4A). To investigate the influence of innate immune cell-derived IFN-γ on the activation of myeloid cells within secondary lymphoid organs, we analyzed splenocytes from VACV-infected mice. VACV-infected mice of the analyzed genotypes showed significantly increased percentages as well as enhanced total counts of PMN in the spleen when compared with uninfected controls (Fig 4B). In contrast, the percentages of macrophages decreased in the spleen of WT and IFN-γ^Ncr1-ON^ mice when compared with uninfected controls, while in IFN-γ^OFF^ mice the percentages of macrophages were unaffected (Fig 4C). Of note, upon infection total cell numbers of macrophages remained stable in all analyzed mice (Fig 4C). These data indicate that IFN-γ expression only by Ncr1^+^ cells can affect PMN numbers in blood and spleen. Of note, in VACV-infected IFN-γ^OFF^ mice MHC-II expression on macrophages was significantly downregulated, while macrophages from WT and IFN-γ^Ncr1-ON^ mice showed comparable MHC-II expression (Fig 4D). Thus, upon VACV infection IFN-γ expression by Ncr1^+^ cells is sufficient to confer protection. Moreover, IFN-γ responses of Ncr1^+^ cells modulate macrophage activation and myeloid cell function.

## Discussion

VACV encodes several modulators of host immunity, including the viral soluble IFN-γ receptor B8 and inhibitors of STAT-1 signaling, such as the viral phosphatase H1 and VH1 [36–38]. Nevertheless, the IFN-γ axis is still critically needed in order to protect mice against lethal VACV infection [17, 18]. Here we focused on the analysis of IFN-γ that is expressed by innate immune cells during homeostasis and VACV infection. To this end, we generated conditional IFN-γ^OFF^ mice in which the IFN-γ gene function can be reconstituted in a Cre-dependent manner. By intercrossing such IFN-γ^OFF^ mice with CD4-Cre we obtained IFN-γ^CD4-ON^ mice in which the IFN-γ gene function was reconstituted primarily in T cells, but also to a lesser extent in some other cell subsets. The issue of cell type-specific gene targeting in Cre mice, including CD4-Cre mice, has recently been addressed inter alia in letters by Reizis and Becher [39, 40]. Correspondingly, CD4-Cre mice are not suitable to reconstitute *Ifng* gene function specifically in T cells and therefore experiments with IFN-γ^CD4-ON^ mice are not appropriate to further dissect the contribution of IFN-γ responses of different cell subsets. In contrast, IFN-γ^Ncr1-ON^ mice that show IFN-γ expression in innate immune cells such as NK cells were as resistant to VACV infection as WT controls, whereas Cre-negative littermates succumbed to the infection with similar kinetics as IFN-γ^-/-^ mice. These data indicate that early IFN-γ responses by Ncr1^+^ cells are sufficient to establish an anti-viral cytokine milieu, to balance myeloid cell numbers, to control viral replication, and to promote the induction of an overall protective immune response, even when the IFN-γ gene reconstitution efficacy is below 100%.

Previous studies with mice lacking either IFN-γ or the IFN-γ receptor revealed the critical role of the IFN-γ axis during VACV infection [17, 18]. Moreover, a vast array of genetically modified mice lacking either NK cells, T cells, or other cell subsets, which consequently were also devoid of the corresponding effector functions, including IFN-γ and perforin, showed enhanced susceptibility to VACV infection [10–15]. More recent studies showed that IFN-γ^-/-^ mice were protected against lethal VACV infection upon adoptive transfer of IFN-γ competent CD8^+^ T cells, highlighting the critical role of T cell-derived IFN-γ during VACV infection [18]. Nevertheless, the physiological role of the IFN-γ responses derived from innate immune cells was not well understood, yet. Adoptive transfer experiments are generally informative, but sometimes they are difficult to interpret due to (i) the manipulation of the cell subsets of interest during the purification procedure, (ii) potentially non-physiological homing properties of adoptively transferred cell subsets, and (iii) very low numbers of transferred cells homing to the relevant sites. Therefore, we adapted a genetic approach in which the endogenous *Ifng* gene function can be cell-selectively reconstituted upon Cre expression. To this end, by homologous recombination we introduced a loxP flanked translational and transcriptional stop (TRAP) cassette into the first intron of the IFN-γ gene of embryonic stem cells. Following Cre mediated deletion of the TRAP cassette the gene function is reconstituted without leaving a residual loxP site in the promoter region. Upon *in vitro* stimulation, splenocytes from IFN-γ^Ncr1-ON^ mice showed IFN-γ expression primarily in NK cells, while WT mice showed IFN-γ expression in NK and T cells.

Similar to WT mice, also VACV-infected IFN-γ^Ncr1-ON^ mice showed two waves of IFN-γ expression in the serum. The first wave was moderately reduced and the IFN-γ peak on 4 dpi was completely absent. Nevertheless, such mice survived VACV infection. These results indicated that to a large extent also Ncr1^+^ cells contribute to early IFN-γ, whereas they produce IFN-γ only in the beginning of the second wave, and still confer protection from VACV infection. ILCs, NKT cells, and γδ T cells also have been reported to express IFN-γ early during viral infection and therefore could additionally contribute to the first wave of IFN-γ expression. Amongst ILC, ILC1 and ILC3 can express Ncr1 and are able to produce IFN-γ [41]. Importantly, ILC3 are primarily localized in gut mucosa and interact with microbiota. In contrast, ILC1 have previously been shown to include NK cells and to be involved in viral infections [42, 43]. We found that in IFN-γ^Ncr1-ON^ mice more than 50% of conventional NK cells show *Ifng* reconstitution, whereas non-NK ILC1 are very rare in the liver and show very low levels of recombination. Formally, we cannot exclude that a minor subset of non-NK ILC1 showed IFN-γ reconstitution. Nevertheless, because non-NK ILC1 are a very minor cell subset, in VACV infected IFN-γ^Ncr1-ON^ mice the amount of IFN-γ contributed by non-NK ILC1 at best can be only very marginal when compared with the contribution of classical NK cells. Of note, *ex vivo* isolated liver-resident NK cells showed significantly reduced IFN-γ expression upon *in vitro* stimulation when compared with conventional NK cells.

IFN-γ is known to regulate the magnitude and composition of cytokine and chemokine responses [44]. Therefore, it was surprising that IFN-γ reconstitution in Ncr1^+^ cells sufficed to induce a sustained anti-viral cytokine milieu following VACV infection. Upon VACV infection of IFN-γ^OFF^ mice the cytokines IL-12p70 and IL-18 that activate NK cells to produce IFN-γ were deregulated, i.e., IL-12p70 was enhanced and IL-18 was reduced when compared with WT controls. IFN-γ^OFF^ mice also showed increased G-CSF serum levels and accordingly enhanced numbers of granulocytes in the periphery. This is in accordance with other studies showing that granulocyte egress from the BM is induced by G-CSF, which in turn is inhibited by IFN-γ [45, 46]. Furthermore, it was shown that PMN development is prevented by IFN-γ [45, 47, 48]. In accordance, in VACV infected WT mice that mount systemic IFN-γ expression, we observed a reduced abundance of PMN in blood, when compared with VACV infected IFN-γ^OFF^ mice. Thus, NK cell-derived IFN-γ contributes to modulate PMN immunity.

It was discovered earlier that NK cells need to be in close proximity to, or even in direct contact with, myeloid cells in order to get activated and to mount IFN-γ responses that in turn shape myeloid cell function [49–53]. This model is supported by our data, showing that NK cell-derived IFN-γ is necessary to prevent the earlier published capacity of VACV to downregulate MHC-II expression on macrophages [54–56]. Previous studies already indicated that T cells were major producers of the second IFN-γ wave after VACV infection and that the presence of IFN-γ expressing CD8^+^ T cells was of key relevance to control the infection [15, 18, 57]. Nevertheless, we found that Ncr1-specific reconstitution of the IFN-γ gene function was sufficient to confer activation of the myeloid cell compartment upon VACV infection and to control the infection.

IFN-γ has broad modulatory effects on immune as well as non-immune cells [58]. Interestingly, excessive NK cell responses may impair adaptive immunity and attenuate the induction of memory responses, e.g. after vaccination or during chronic infection [59–65]. On the other hand, patients with NK cell deficiencies or deficiencies in the IFN-γ axis are predisposed to severe, recurrent mycobacterial as well as viral infections [66–69]. Sepsis can induce severe and fatal immunoparalysis in patients. IFN-γ can restore leucocyte function in such patients by reversing sepsis-induced defects in glycolysis and oxidative metabolism [70–72]. Furthermore, several previous reports highlighted the possibility to use IFN-γ as an adjuvant in vaccination approaches [73–75], and NK cells have been implicated to play a critical role early after stem cell transplantation [76–78]. Finally, NK cells can also be effectors in acquired immunity [79–81]. Thus, NK cell-mediated immunity and the IFN-γ axis are interesting targets that need to be better understood for exploitation in clinical applications. We found that the induction of early IFN-γ expression by Ncr1^+^ cells is critical for eliciting fully effective immune responses against VACV infection. These findings clearly demonstrate the underestimated importance of NK cell-derived IFN-γ and give rise to new concepts on how protective immunity is shaped.

## Material and methods

### Ethics statement

All animals were handled in compliance with regulations of the German Animal protection law (Tierschutzgesetz). Experiments were approved by the Niedersächsisches Landesamt für Verbraucherschutz und Lebensmittelsicherheit (LAVES, Oldenburg, Germany, Grant number 33.12-42502-04-13/1072).

### Mice and viruses

C57BL/6 (WT) (Harlan Winkelmann), B6.FVB-Tg(EIIa-cre)C5379Lmgd (EIIa-Cre^-/+^) [82], B6.Tg(Ncr1-icre)265Sxl (Ncr1-Cre^-/+^) [32], B6.Tg(CD4-cre)1Cwi (CD4-Cre^-/+^) [31], Gt(ROSA)26Sortm1Hjf (R26RFP^OFF^) mice [83], and B6.129P2-Ifng^tm1Uka^ (IFN-γ^OFF^) mice were bred under specific pathogen free conditions at the central animal facility of TWINCORE and the Helmholtz Center for Infection Research (HZI), Brunswick, Germany. Mouse experimental work was carried out using 8 to 12 week old mice in compliance with regulations of the German animal protection law. The VACV strain Western Reserve (originally provided by Bernard Moss, NIH, Bethesda, MD) was propagated on BHK-21 cells (ATCC CCL-10). Virus stocks were purified by sucrose density gradient ultracentrifugation. To determine virus loads, organ homogenates were titrated on RK13 cells (ATCC CCL-37). Organ homogenates were added to a confluent cell layer and overlaid with methylcellulose. Cell were incubated for 48 hours at 37°C and plaque-forming units (pfu) per ml tissue were determined using crystal violet staining. In all infection experiments mice were i.v. infected with 2 x 10^6^ pfu VACV if not otherwise indicated.

### Generation of IFN-γ^Ncr1-ON^ mice

For the generation of IFN-γ^OFF^ mice, a modified loxP flanked translational and transcriptional gene trapping cassette (TRAP) [84, 85] encompassing a loxP flanked neo cassette was introduced into the first intron of the IFN-γ gene of the E14 embryonic SV129/Ola stem cell, subclone IB10 [86]. The TRAP cassette contained a strong engrailed 2 splice acceptor that confers a premature transcriptional stop of the IFN-γ gene, whereas following Cre mediated deletion of the TRAP cassette the *Ifng* gene function is reconstituted with leaving a residual loxP site in the first intron region. The genetically modified embryonic stem cells were microinjected into BALB/c blastocysts and a chimeric founder was identified that upon mating with BALB/c females passed the introduced mutation on to the next generation with the expected frequency. In order to remove the neo cassette *in vivo* without also deleting the TRAP cassette, transgenic offspring were intercrossed with EIIa-Cre mice that confer partial deletion of loxP flanked DNA segments. Indeed, approximately 15% of the offspring showed deletion only of the neo cassette, whereas the TRAP cassette was still present. These mice were back crossed for 4 generations on the C57BL/6 background (>99.6%). Offspring was further back crossed for two more generations and mice with crossing over event in close proximity to the IFN-γ locus (chromosome 10) were identified by using a short tandem repeat (STR) screening speed congenic approach (GVG Diagnostics). These IFN-γ^OFF^ mice were then intercrossed with CD4-Cre^+/-^ or Ncr1-Cre^+/-^ mice in order to obtain IFN-γ^CD4-ON or^ IFN-γ^Ncr1-ON^ mice, respectively.

### Genotyping

Genotyping was performed from ES cells or ear biopsies with primer pairs for floxed or wt IFN-γ locus: IFN-γ^OFF^ (fwd 5’-TTTTGCCAGTTCCTCCAGAT-3’; rev 5’-GCTGGCCCTACTCACACTTC-3’) and for IFN-γ^WT/ON^ (fwd 5’-TTTTGCCAGTTCCTCCAGAT-3’; rev 5’- TCAGAGGCCTGGACCATAAG-3’).

### Cytokine and chemokine analyses

Serum was tested for IFN-γ using the Ready**-**SET**-**Go! Kit (eBioscience), following the manufacturer’s instructions. Multiplex cytokine array was performed using the Bio-Plex Pro Mouse Cytokine 23-Plex Assay (Bio**-**Rad), following the manufacturer’s instructions.

### Cell isolation and flow cytometry

Splenocytes were filtered through 70 μm cell strainers and centrifuged at 300 g for 6 min at 4°C. Myeloid cells from liver were prepared as described previously [19]. Following red blood cell lysis (Sigma), cells were immunolabeled with fluorochrome-conjugated antibodies (S1 Table) (Biolegend and BD) for flow-cytometry analysis (LSR II Sorb, Becton Dickinson). Intranuclear staining of ILC1 was performed using the Transcription Factor Buffer Set (BD Pharmingen). Blood was directly immunolabeled with fluorochrome-conjugated antibodies (S1 Table) (Biolegend and BD) for flow-cytometry analysis (LSR II Sorb, Becton Dickinson) and subsequently treated with red blood cell lysing solution (BD). The gating of different cell populations is indicated in the supporting information section (S2 Fig). Cell counts were determined using AccuCheck counting beads (Life technology).

### Stimulation of splenocytes and intracellular cytokine staining

Isolated splenocytes of naïve mice were stimulated with 10 ng/ml PMA and 1 μg/ml ionomycin, splenocytes of VACV-infected mice were re-stimulated with CD3/CD28 T cell activation beads or left untreated for 4 hours in the presence of Golgi-Block (BD). Cells were immunolabeled with fluorochrome-conjugated antibodies (Biolegend and BD, see S1 Table) and subsequently stained with an intracellular cytokine kit (BD) for flow-cytometry analysis (LSR II Sorb, Becton Dickinson).

### Statistical analysis

Statistical analyses were performed using GraphPad Prism 6 software. Heatmaps were generated from mean values of each parameter using Prism 7.

## Supporting information

S1 Fig Ncr1-specific targeting of ILC1 and IFN-γ production of conventional and resident NK cells. (A) Lymphocytes were isolated from liver of R26RFP^Ncr1-ON^ mice and analyzed by flow cytometry. Percentage of RFP^+^ cells of either Ncr1^+^NK1.1^+^ NK cells or Lin^-^CD127^+^T-bet^+^Rorγt^-^ ILC1 were determined (n = 2, N = 1). (B)/(C) Lymphocytes were isolated from liver of WT, IFN-γ^OFF^ and IFN-γ^Ncr1-ON^ mice, *in vitro* stimulated with PMA/ionomycin for 4 h, and then analyzed by flow cytometry. Percentage of IFN-γ^+^ cells of either Ncr1^+^NK1.1^+^CD49b^+^ conventional NK cells or Ncr1^+^NK1.1^+^CD49a^+^ resident NK cells were determined (n = 4, N = 2); paired T Test. Error bars indicate mean ± SEM; ***p ≤ 0.001, **p ≤ 0.01, *p ≤ 0.05.
(TIF)

S2 Fig Gating strategy for the analysis of defined cell subsets. Cells from spleen or liver were isolated as described. (A) Amongst CD3^-^CD19^-^ negative cells and F4/80^+^ macrophages and Ly6G^+^ polymorphonuclear neutrophils (PMN) were analyzed. (B) Amongst NK1.1^-^ and TCRγδ^-^ cells, CD3^+^CD4^+^ T cells were analyzed. Amongst CD3^-^CD4^-^ cells NK1.1^+^Ncr1^+^ NK cells were analyzed. (C) ILC1 were defined as lineage^-^CD127^+^T-bet^+^RORγt^-^ Ncr1^+^ cells.
(TIF)

S1 Table List of antibodies used in this study with clones, fluorophores, and manufacturers. (XLSX)
