# Supplementary figures and images for: Selective reconstitution of IFN‑γ gene function in Ncr1+ NK cells is sufficient to control systemic vaccinia virus infection
Source: PLoS Pathog. 2020 Feb 5;16(2):e1008279. doi: 10.1371/journal.ppat.1008279 (PMC7028289; doi:10.1371/journal.ppat.1008279)

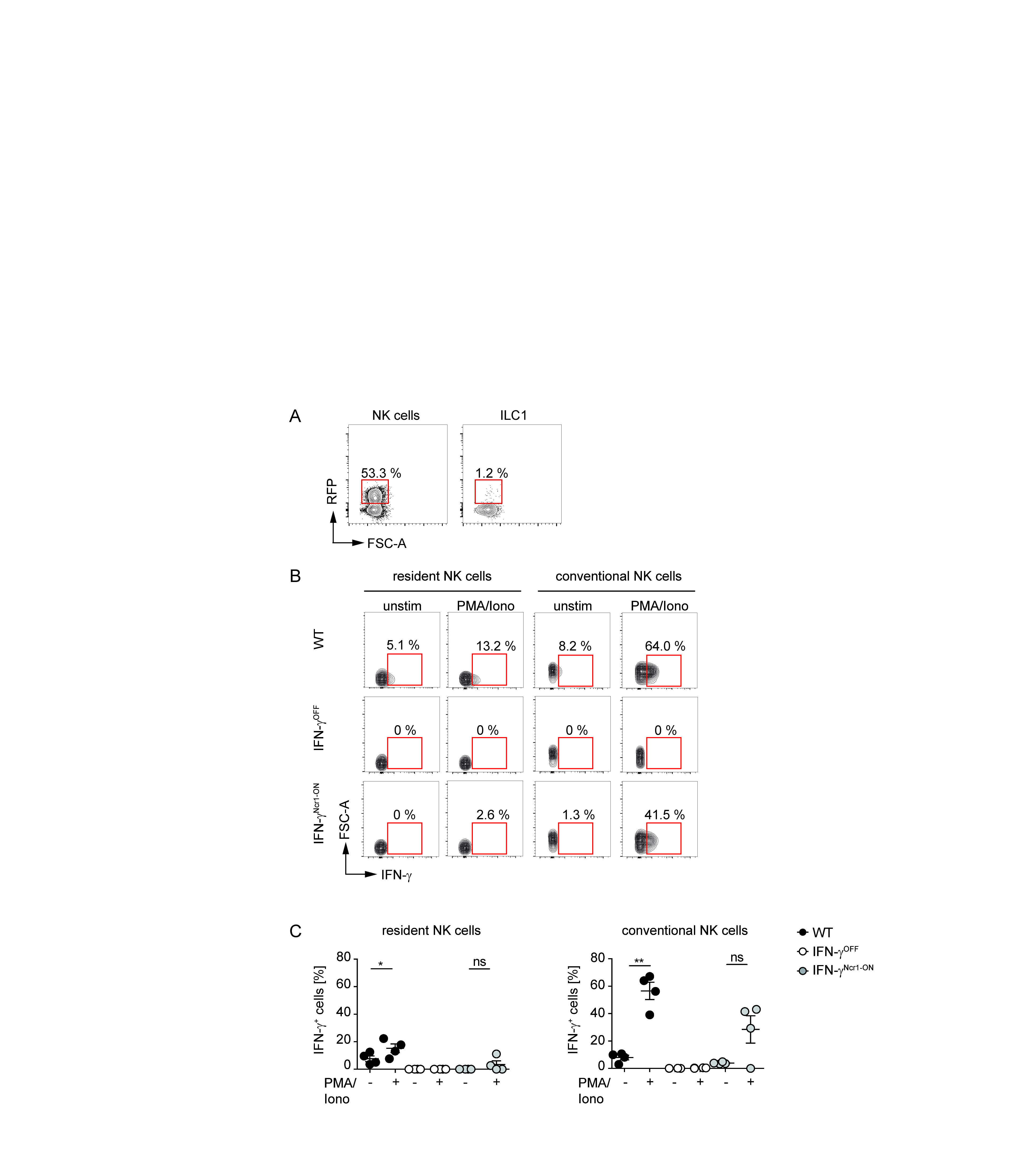

Supplement: S1 Fig — (A) Lymphocytes were isolated from liver of R26RFPNcr1-ON mice and analyzed by flow cytometry. Percentage of RFP+ cells of either Ncr1+NK1.1+ NK cells or Lin-CD127+T-bet+Rorγt- ILC1 were determined (n = 2, N = 1). (B)/(C) Lymphocytes were isolated from liver of WT, IFN-γOFF and IFN-γNcr1-ON mice, in vitro stimulated with PMA/ionomycin for 4 h, and then analyzed by flow cytometry. Percentage of IFN-γ+ cells of either Ncr1+NK1.1+CD49b+ conventional NK cells or Ncr1+NK1.1+CD49a+ resident NK cells were determined (n = 4, N = 2); paired T Test. Error bars indicate mean ± SEM; ***p ≤ 0.001, **p ≤ 0.01, *p ≤ 0.05. (TIF) [file ppat.1008279.s001.tif]

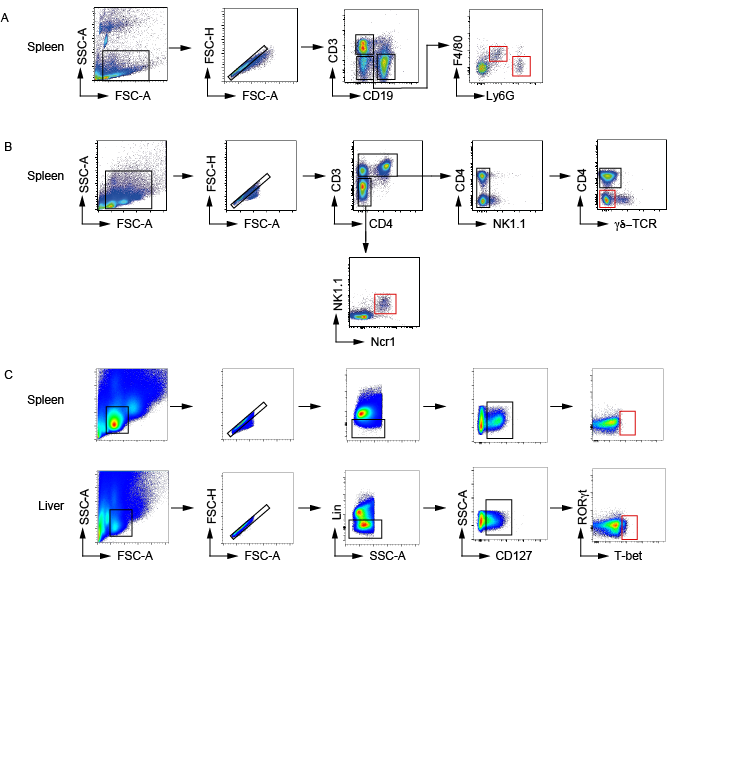

Supplement: S2 Fig — Cells from spleen or liver were isolated as described. (A) Amongst CD3-CD19- negative cells and F4/80+ macrophages and Ly6G+ polymorphonuclear neutrophils (PMN) were analyzed. (B) Amongst NK1.1- and TCRγδ- cells, CD3+CD4+ T cells were analyzed. Amongst CD3-CD4- cells NK1.1+Ncr1+ NK cells were analyzed. (C) ILC1 were defined as lineage-CD127+T-bet+RORγt- Ncr1+ cells. (TIF) [file ppat.1008279.s002.tif]
